# Observed and personally experienced discrimination: findings of a cross-sectional survey of physicians and nursing staff

**DOI:** 10.1186/s12960-022-00779-0

**Published:** 2022-12-09

**Authors:** Arda Yolci, Liane Schenk, Pia-Theresa Sonntag, Lisa Peppler, Meryam Schouler-Ocak, Anna Schneider

**Affiliations:** 1 Institute of Medical Sociology and Rehabilitation Science, Charité-Universitätsmedizin Berlin, Corporate Member of Freie Universität Berlin and Humboldt-Universität zu Berlin, Charitéplatz 1, 10117 Berlin, Germany; 2 Department of Psychiatry and Psychotherapy, Charité-Universitätsmedizin Berlin, Corporate Member of Freie Universität Berlin and Humboldt-Universität zu Berlin, Berlin, Germany; 3 Department of Psychiatry and Psychotherapy, Psychiatrische Universitätsklinik der Charité im St. Hedwig-Krankenhaus, Berlin, Germany

**Keywords:** Discrimination, Healthcare staff, Hospital, Ethnicity, Migration, Cultural competence, Germany

## Abstract

**Background:**

Discrimination against hospital staff based on ascribed features is prevalent in healthcare systems worldwide. Detrimental effects on health and quality of patient care have been shown. Our study aims to describe and analyse the discrimination experiences of both physicians and nurses, specifically for the German hospital context.

**Methods:**

A cross-sectional online survey on observed and personally experienced discrimination at work addressed staff from 22 hospitals of two organizations in Germany. Sociodemographic and occupational as well as institutional characteristics served as independent variables. In multivariable analyses, block- and stepwise logistic regressions were calculated for the two dependent variables (witness and victim of discrimination). Sensitivity analyses with imputed data for missings were performed.

**Results:**

*N* = 800 healthcare professionals (*n* = 243 physicians, *n* = 557 nurses; response rate: 5.9%) participated in the survey. 305 respondents (38.1%) were witnesses of discrimination, while 108 respondents (13.5%) were victims of discrimination in their wards. Reasons for observed discriminatory acts were predominantly attributed to the ethnicity of the person concerned, their appearance and language, whereas personally affected staff most frequently cited gender as a reason, followed by ethnicity, and physical appearance. In multivariable models, cultural competence significantly increased the likelihood of witnessing discrimination (*β* = .575; *p* = .037). In terms of the likelihood of being a victim of discrimination, in addition to cultural competence (*β* = 2.838; *p* =  < .001), the interaction of the effects of gender and professional group was statistically significant (*β* = .280; *p* = .010).

**Conclusions:**

Given the extent of experienced and observed discrimination, appropriate institutional responses are needed. Further research on discriminatory structures in the German-speaking health care system should focus on discrimination at the intersection of ethnicity, gender and occupation.

**Supplementary Information:**

The online version contains supplementary material available at 10.1186/s12960-022-00779-0.

## Background

Discrimination against persons based on characteristics linked to their ethnicity or migration status is prevalent worldwide [1]. In Germany, too, representative longitudinal studies of the population documented a general polarization of society and the emergence of extreme right-wing and nationalist tendencies [2]. We understand discrimination to entail people being disadvantaged and belittled in relation to ascribed features, such as, e.g., gender, social background, age, disability and sexual orientation, as well as religion, language and ethnic origin [3, 4]. Discrimination mechanisms are anchored on the individual and institutional levels and in society as a whole [4]. Numerous systematic reviews documented the negative effects on the mental and physical health of those affected, such as increased prevalence of depression, anxiety disorders and excess weight [5, 6].

The health system mirrors the state of society and, therefore, also reflects existing discrimination dynamics that may impact both patients and staff. Relevant studies in the hospital environment previously examined different professions and clinical specialties [7, 8] and certain forms of discrimination, e.g., regarding the gender of affected persons [9–11]. Studies focusing on systematic discrimination against healthcare staff based on features associated with their ethnicity, such as, e.g., nationality, language or religion, showed that in countries of the Global North, e.g., the USA, the UK and Canada, racial discrimination in their profession is an everyday experience for nurses [12, 13] and physicians [14–16] and that such discrimination has a continuous history. Forms of discrimination included disadvantageous treatment on both interpersonal and institutional levels, e.g., more difficult career progression [17–19], more frequent disciplinary procedures [20], more unpaid overtime and less participation in work planning [21] and most recently in relation to the COVID-19 (coronavirus disease 2019) pandemic, indications of higher rates of mortality and exposure to infection [22]. Studies pointed to a wide spectrum of persons, e.g., patients, colleagues and superiors, as the source of reported discrimination [23–25]. A clustering of discrimination experiences linked to precarious forms of employment such as part-time work [10, 21] and the educational level of those affected [7, 26] was further observed. In addition to well-known effects of discrimination on individual health and well-being, additional professional and institution-related impacts of such experiences in the workplace were widespread. Discrimination and threats at the workplace were associated with lower job satisfaction [13], poorer mental and physical health [7, 27], higher stress levels [8], more days lost through sickness and more frequent mental and physical withdrawal [23, 28, 29]. Persons affected by discrimination reported diminished self-esteem and reduced productivity [30, 31]. Studies also showed that in terms of institutional outcomes, discrimination was related to staff fluctuation of both nurses and physicians [13, 32]. Direct and indirect effects of discrimination in clinical workplaces were further associated with negative impacts on the quality of patient care and higher costs in the healthcare system [8, 28, 31].

Studies on discrimination of healthcare staff demonstrated the importance of taking into account the interplay and mutual reinforcement of discrimination mechanisms based on characteristics of the affected persons, such as social background, gender, professional status, age and ethnicity or experience of migration [16, 30, 33, 34]. This so-called intersectional approach is highly relevant in analysing discrimination both on the micro level and in institutional structures, e.g., in a hospital, where hierarchical structures and a high proportion of women (particularly in nursing) prevail [8, 17, 33]. Racism and discrimination against persons due to their nationality, ethnicity or migration status followed specific historical lines of development on the national level [35]. It is, therefore, important to take into account the particular national situation and its distinctive features from other contexts. In Germany, the early loss of its colonies after the first World War, the extermination of ethnic minorities during the Nazi regime, and a high influx of migrant workers and their families from Turkey, the former Yugoslavia and Southern European countries such as Greece and Portugal in the 1950´s to 1970´s have led to a different formation of minorities in comparison with, e.g., the USA [36]. Despite some qualitative studies in German-speaking countries, in comparison with the state of international research there are large gaps in quantitative research in Germany on discrimination experienced by hospital healthcare staff. Large-scale studies in Germany are thus required relating to the two numerically largest groups of staff in the healthcare field.

Accordingly, an online survey of discrimination experiences addressing hospital nurses and physicians was carried out as part of a larger multicenter mixed-methods research project. Our study was the first to focus on (a) the description of healthcare staff’s observed or personally experienced discrimination in the workplace and of identified perpetrators and ascribed reasons, and (b) the examination of interpersonal and institutional factors associated with these discrimination experiences of healthcare staff.

## Methods

### Data collection

Between May and November 2018, healthcare staff in 22 hospitals run by two organizations participated in a standardized online survey. The study received the approval of the relevant ethics committee. All staff members who were active as nurses or physicians in the hospitals run by the two organizations at the time of the survey were eligible to participate. The invitation to participate in the survey was sent by email via a personalized link to the work address of all physicians and nurses, and where no email account was available, by a QR (quick response) code sent by mail with the pay slip. Unipark software was used to administer the survey. The time required to answer the questionnaire was approximately 15 min. The aim was to complete a full census of both organizations with approximately 3700 physicians and 9800 nurses. No additional demographic information was available on the total population for non-responder analysis. To increase the response rate, we sent reminders and an additional attempt with written surveys was carried out among the physicians in one of the organizations.

### Measures

The two dependent variables, observed and personally experienced discrimination, were formulated on the basis of the questionnaire "Discrimination experiences of migrants in Germany" developed in the context of a survey by the integration agencies of the intercultural migrant center IMAZ e.V. (Interkulturelles Migrantenzentrum e.V.) and the German Red Cross regional chapter in Düsseldorf. The survey addresses with two separate questions whether the respondent had ever been a witness to or victim of discrimination at their own wards. If the answer was yes, based on the dependent variable, further questions followed regarding information on the person being discriminated against, possible reasons for the discrimination, and the perpetrator of the observed or personally experienced discrimination. A set of possible answer categories was provided for each item, supplemented by the category “Other” with a free text field. Multiple entries from the available categories were possible. In addition, two items examined institutional responses to the discrimination, i.e., discussion of the incident or implementation of measures derived from it in the department. Further questions covered additional variables, i.e., sociodemographic characteristics of the participants, such as age, gender, and migration background. Respondents were allocated to one of three categories in relation to their migration background: no migration background, migration background on one side, or migration background on both sides, based on the country of their parents’ birth. In addition, data on employment, i.e., professional group, type of employment and working hours, were included. Institutional characteristics reflected the estimated percentage of staff and patients with a migration background in the respondents’ departments as well as the affiliation to one of the two surveyed organizations (anonymized in organisation A and B due to data protection requirements). As a relevant influencing factor in relation to discrimination, respondents’ cultural competence was also examined using a translated version of the validated Short Form Cultural Intelligence Scale [37]. The translated items of variables used in this study are available in Additional file 1.

### Statistical analyses

Variables are described with frequency distributions or with mean values and standard deviations according to the scales of measurement. In bivariate analyses, Pearson Chi-square tests and Student’s *t* tests were conducted to examine differences in the distribution of categorical and metrical variables, respectively, in respondents with and without any discrimination experiences. In multivariable analyses, logistic regressions were calculated in which, in two separate models, potential influencing variables were placed block- and stepwise, in relation to the two dependent variables, i.e., observed and personally experienced discrimination. In the first regression step in each model, the effect of sociodemographic and employment factors on the outcome was examined. In the second step, cultural competence and institutional features were added for a joint model. To examine potentially linked effects of sociodemographic and employment factors, an interaction term was created from the variables professional group and gender; this term was included in the regression models in addition to the relevant individual variables. In sensitivity analyses, regression models for both dependent variables were repeated with an imputed data set which was created using the MULTIPLE IMPUTATION procedure in SPSS. Range restrictions were carried out for four variables in the imputation process, including age (min = 18, max = 100), proportion of staff or patients with a migration background (min = 0, max = 100) and cultural competence (min = 1, max = 5). The statistical evaluation was carried out using version 25 of IBM SPSS Statistics.

## Results

The final sample comprised *N* = 800 staff members of the participating institutions (response rate: 5.9%). Respondents’ characteristics are shown in Table 1. Altogether, 305 respondents (38.1%) stated that they had witnessed and 108 respondents (13.5%) stated that they had personally experienced discrimination in their department. For observed events, affected persons were mostly colleagues (*n* = 257), and patients (*n* = 211; see Table 2). Stated reasons were the ethnicity of the discriminated person (*n* = 196), their appearance (*n* = 175) or language (*n* = 145). Meanwhile, of those participants who had experienced discrimination themselves, the majority reported gender (*n* = 62) as the reason for the event, followed by ethnicity (*n* = 28) and appearance (*n* = 27; see Table 2). Witnesses to and victims of discrimination both reported that the discriminating actors most frequently were patients (81.3% and 67.6%, respectively), although colleagues (55.4% and 36.1%) and superiors (20.3% and 33.3%) were also named. Out of all cases of observed or personally experienced discrimination (*n* = 320), 39.1% of respondents (*n* = 125) stated that antidiscrimination measures were subsequently discussed in their department. However, only 27.8% of respondents (*n* = 89) stated that relevant measures were implemented.

**Table 1:** Description of respondents (all and categorized by status of discrimination experiences) and institutional characteristics

Variable	All respondents (*n* = 800) *n* (%)/mean (SD)	Witness of discrimination (*n* = 305) *n* (row %^a^)/mean (SD)	Victim of discrimination (*n* = 108) *n* (row %^a^)/mean (SD)	No discrimination experience (*n* = 480) *n* (row %^a^)/mean (SD)
Institution				
Organization A	271 (33.9%)	113 (41.7%)	51 (18.8%)	151 (55.7%)
Organization B	529 (66.1%)	192 (36.3%)	57 (10.8%)*	329 (62.2%)
Gender				
Male	241 (31.0%)	94 (39.0%)	35 (14.5%)	141 (58.5%)
Female	536 (69.0%)	201 (37.5%)	69 (12.9%)	326 (60.8%)
Professional group				
Physicians	243 (30.4%)	101 (41.6%)	45 (18.5%)	137 (56.4%)
Nursing staff	557 (69.6%)	204 (36.6%)	63 (11.3%)*	343 (61.6%)
Migration background				
No migration background	621 (78.2%)	235 (37.8%)	78 (12.6%)	375 (60.4%)
Migration background on one side	56 (7.1%)	24 (42.9%)	11 (19.6%)	30 (53.6%)
Migration background on both sides	117 (14.7%)	45 (38.5%)	19 (16.2%)	70 (59.8%)
Employment contract				
Fixed-term	207 (26.4%)	93 (44.9%)	34 (16.4%)	111 (53.6%)
Permanent	578 (73.6%)	207 (35.8%)*	74 (12.8%)	359 (62.1%)*
Work time model				
Full time	492 (62.2%)	192 (39.0%)	69 (14.0%)	290 (58.9%)
Part time	299 (37.8%)	110 (36.8%)	38 (12.7%)	184 (61.5%)
Age (in years)	41.3 (11.1)	40.3 (10.4)*	39.4 (10.2)	41.9 (11.4)*
Cultural competence	3.5 (0.6)	3.6 (0.6)*	3.8 (0.5)*	3.4 (0.6)*
Estimated proportion of staff with migration background in respondent’s own department (in %)	22.1 (17.5)	22.5 (16.8)	22.4 (16.9)	21.8 (17.8)
Estimated proportion of patients with migration background in respondent’s own department (in %)	30.5 (20.5)	32.2 (20.3)	33.0 (22.2)	29.1 (20.4)*

^a^Row percentages for witnesses and victims of discrimination and those without discrimination experiences describe the relative amount of counts in the respective total of the row variable; Pearson Chi-square tests and Student’s *t* tests were conducted to examine differences in the distribution of categorical and metrical variables, respectively. Statistically significant differences (*p* ≤ 0.05, two-sided) in distributions between respondents with and without discrimination experiences at their wards are denoted by an asterisk (*)

**Table 2:** Frequency of observed and personally experienced discrimination and characteristics associated with the discrimination event

Characteristics of discrimination	Dependent variables	
	Witness to discrimination *n* (%^a^)	Target of discrimination *n* (%^a^)
Target of discrimination		
Colleague	257 (84.3)	–
Patient	211 (69.2)	–
Superior	21 (6.9)	–
Other	5 (1.6)	–
Reason for discrimination		
Ethnicity	196 (64.3)	28 (25.9)
Appearance	175 (57.4)	27 (25.0)
Language	145 (47.5)	11 (10.2)
Religion	125 (41.0)	15 (13.9)
Gender	109 (35.7)	62 (57.4)
Age	35 (11.5)	24 (22.2)
Other	23 (7.5)	20 (18.5)
Source of discrimination		
Patient	248 (81.3)	73 (67.6)
Colleague	169 (55.4)	39 (36.1)
Superior	62 (20.3)	36 (33.3)
Other	16 (5.2)	3 (2.8)

^a^Proportion of answers in which discrimination was reported (*n* = 305 for observed discrimination, *n* = 108 for personally experienced discrimination)

Bivariate analyses revealed that witnesses in comparison with non-witnesses of discrimination were statistically more likely to be fixed-term employees, to be younger, and to have higher cultural competence. Victims of discrimination in comparison with non-victims were more often employed in organization A, were more often physicians, and had higher cultural competence. Those participants who reported no discrimination experiences at their wards were statistically more likely to be permanent employees, to be older, to have lower cultural competence and to work in wards with fewer patients with migration background than participants who reported any discrimination experience (see Table 1).

The findings of the multivariable stepwise logistic regression analyses showed that the relative probability of observing a discrimination event was significantly associated only with respondents’ cultural competence (see Table 3). According to this, respondents with greater cultural competence showed a higher relative probability of observing discrimination in their department (*β* = 1.583; *p* = 0.002). All other sociodemographic, employment and institutional features were not significantly linked to this dependent variable. The statistical model was significant (*χ*^2^(12) = 23.11; *p* = 0.027; *N* = 734). Concerning features associated with the dependent variable of personally experienced discrimination,  three variables retained stable effects throughout the stepwise structure (see Table 3): first, the relative probability of experiencing discrimination in the respondent’s own department was significantly lower in one of the two organizations (*β* = 0.575; *p* = 0.037). In addition, the interaction of the effects of gender and professional group was statistically significant (*β* = 0.280; *p* = 0.010), so that male nurses and female physicians had a higher probability of experiencing discrimination than female nurses and male physicians. Greater cultural competence of the respondents also increased the relative probability of reporting personal experience of discrimination (*β* = 2.838; *p* =  < 0.001). The statistical model for the second outcome was also significant (*χ*^2^(12) = 51.32; *p* < 0.001; *N* = 734).

**Table 3:** Results of stepwise logistic regressions for the dependent variables ‘witness of discrimination’ and ‘victim of discrimination’

Variable	Outcome: witness of discrimination						Outcome: victim of discrimination					
	First step			Second step			First step			Second step		
	Beta (*β*)	95% CI	*p* value	Beta (*β*)	95% CI	*p* value	Beta (*β*)	95% CI	*p* value	Beta (*β*)	95% CI	*p* value
Gender: Female	1.233	.709; 2.143	.458	1.128	.645; 1.975	.673	2.009	.947; 4.262	.069	1.702	.786; 3.683	.177
Age	.991	.976; 1.006	.219	.994	.979; 1.009	.431	.979	.957; 1.001	.060	.983	.961; 1.006	.148
Migration background: on one side	1.245	.682; 2.271	.475	1.168	.636; 2.144	.617	1.387	.640; 3.006	.407	1.275	.577; 2.816	.548
Migration background: on both sides	.900	.584; 1.388	.635	.781	.497; 1.227	.283	1.185	.657; 2.136	.573	.897	.486; 1.655	.727
Institution: organization B	.820	.578; 1.162	.265	.916	.633; 1.327	.644	**.532**	**.328; .861**	**.010**	**.575**	**.343; .967**	**.037**
Professional group: nurses	1.360	.761; 2.429	.299	1.275	.709; 2.290	.417	1.532	.671; 3.496	.311	1.346	.582; 3.110	.487
Interaction: professional group*gender	.603	.304; 1.193	.146	.644	.322; 1.287	.213	**.236**	**.092; .602**	**.003**	**.280**	**.107; .734**	**.010**
Employment contract: permanent	.791	.519; 1.204	.274	.804	.526; 1.230	.315	1.534	.826; 2.852	.176	1.640	.874; 3.076	.123
Work time model: part time	1.084	.778; 1.511	.632	1.089	.779; 1.523	.619	1.252	.782; 2.005	.349	1.242	.767; 2.012	.379
Proportion of staff with MB in department	–	–	–	.997	.987; 1.007	.556	–	–	–	1.000	.986; 1.014	.962
Proportion of patients with MB in department	–	–	–	1.006	.997; 1.015	.183	–	–	–	1.001	.989; 1.014	.851
Cultural competence	–	–	–	**1.583**	**1.184; 2.118**	**.002**	–	–	–	**2.838**	**1.825; 4.413**	** < .001**

Two separate block- and stepwise regression models were calculated for the dependent variables ‘witness of discrimination’ and ‘victim of discrimination’, respectively. In the first regression step, associations of sociodemographic and employment factors of participants with dependent variables were examined. In the second regression step, individual cultural competence and the proportion of staff and patients with migration background were added to the final model. *N* = 734; *CI* confidence interval, *MB* migration background; significant regression coefficients and respective confidence intervals are in bold print

## Discussion

This study is the first empirical quantitative examination of discrimination experiences among a multi-professional sample of hospital staff in Germany. It addressed a sample of physicians and nurses to determine the prevalence of discrimination and the links to individual and institutional features. Our findings indicate that a substantial proportion of hospital staff observe or personally experience discrimination at their workplace. Cultural competence proved to be a determining factor in identifying discrimination of third parties or personally experienced discrimination. Professional groups that are traditionally underrepresented in healthcare, such as male nurses and female physicians, experience discrimination more frequently, whereas features of the organization also proved to be a determining factor.

### Prevalence and forms of discrimination

Over a third of the physicians and nurses who responded to our survey stated that they had witnessed discrimination in their own department, while 13.5% reported discrimination that they themselves had experienced. Our findings suggest that discrimination, particularly in the context of migration and ethnicity, is just as relevant and topical an issue in German hospitals as described in other international settings [12, 14]. In line with current international research, our study shows that discrimination is perpetrated by patients as well as colleagues and superiors in the hospital setting. Patients are thus the most frequent source of discrimination, which coincides with study findings from the UK and the USA [7, 13, 23]. Since patients are numerically the largest group in the health system, the dominance of discrimination by patients is not surprising. However, the large proportion of discrimination by colleagues and superiors is alarming, particularly since studies showed that this type of discrimination is more stressful for victims than that coming from patients [13, 26]. A wide range of ascribed reasons for discrimination is seen, both observed and personally experienced, while the distribution differs between the two types of discrimination. The ethnicity or appearance of a person were the most frequently mentioned reasons for observed discrimination, whereas gender was pointed out in first place as the reason for personally experienced discrimination. This divergence could be explained by a number of factors. The low number of witnesses of discrimination naming gender as the reason for the discriminatory event could show a general lack of sensitivity when it comes to sexism at the workplace or a normalization and integration of sexist behaviour into day-to-day work dynamics. This could result in unnoticed and overlooked sexist discrimination or a false attribution of reasons for a discriminatory action. For example, when a female nurse wearing a hijab is discriminated against, it may appear to bystanders as if the reason for the act was ‘religion’, when in contrast the nurse herself would possibly say it was because of ‘gender’. While misconceptions in this manner are possible, on the other hand, a bystander could also assume that the discrimination took place because of ‘gender’, when the nurse being discriminated against would possibly argue that her ‘ethnicity’ was the reason. What becomes apparent is the complex nature of intersectional discrimination. Valid study designs and statistical analytic methods to identify reciprocal and multiplying effects between different forms of discrimination are still under development. While only a combination of quantitative and qualitative methods can possibly address the complexity of intersectional discrimination in the health care system, our research shows the multiple ways in which discrimination takes place in German hospitals and should serve as an explorative impulse for further research in this field. Nevertheless, it is striking that observed discrimination was mostly associated with features linked to ethnicity. Although registered separately, ethnicity, religion, language and appearance are not to be understood as separate categories; rather, they are all features that ‘racialize’ a person and classify them as belonging to a group that is perceived as the ‘foreign Other’ by the dominant part of society [3, 26]. Looked at in this way, a considerable proportion of the observed discrimination in our study can be possibly attributed to racist motives. Another difficulty in assessing discrimination in the context of migration in Germany is the underdevelopment of discourse and vocabulary in the field of racism. The prevailing term ‘migration background’ commonly used in government reports and research does not fully capture aspects of ethnicity aside from the migration experience of a person. Only the consideration of multiple aspects of ethnicity, migration experience, socioeconomic status and gender can depict the different kinds of discriminatory dynamics and systems to which persons are subjected.

The fact that far fewer than half of our respondents stated that discrimination events in the department were discussed or relevant measures implemented fits the picture that emerges from studies in Germany as well as in the USA and the UK which locate racism in the health system not only on the interpersonal level but also on the institutional level. This may include a lack of antidiscrimination policies and dedicated operational managers, which may manifest in difficulties for certain population groups when job-hunting [18], unpaid overtime [21] and other forms of institutional discrimination [15, 17, 19]. Our study highlights that both physicians and nurses report insufficient responses to discrimination in German hospitals and a lack of structural change. In light of the rising number of hospital workers with a migration background this is undoubtedly alarming, even more so when taking into account that the majority of the nursing staff in Germany is female [38], while at the same time, our study highlights the high prevalence and ambiguous nature of gender-based discrimination.

### Individual and institutional factors associated with discrimination

Statistical analyses showed a possible interaction effect between profession and gender in our sample. Male nurses stated more often than female nurses that they experienced discrimination, as did female physicians in comparison with male physicians. The fact that female physicians reported discrimination more frequently than male physicians is in line with findings from the USA [7, 40]. Healthcare staff who do not conform to the stereotypical professional image society holds of the male physician and female nurse may be a vulnerable group in relation to discrimination in healthcare [9]. In addition, an institutional effect is revealed that indicates that discrimination may also be associated with the institutional culture and specific measures taken by a hospital. These differences in the prevalence of discrimination between institutions have also been observed in previous studies [7]. However, to qualify this statement we must also point out that it was only the personally experienced discrimination that differed between institutions, whereas there was no difference in the frequency of observed discrimination. Because additional institution-related characteristics were not available for our statistical analyses due to anonymization of the participating organizations, we cannot make any statements about which aspects of organization B may be associated with fewer discrimination events. Regardless, the fact that the institution is a significant factor in experienced discrimination might indicate that there is a possibility and, therefore, potential for reducing such events and creating a safer work environment in single institutions.

Further, multivariable analyses showed that a high level of cultural competence was associated with both observed and personally experienced discrimination. Due to the cross-sectional design of our study, it is not possible to assess causality; however, a link in both directions appears plausible. Healthcare staff with greater cultural competence could be more capable of perceiving and naming discrimination than colleagues who do not have these skills. On the other hand, it may be that people who observe or experience discrimination more frequently develop greater cultural competence through this experience. Using the same sample as in this study, Schenk et al. [41] showed that a positive link exists between healthcare staff’s participation in intercultural training courses and their degree of cultural competence. This finding confirms existing studies on this topic [42, 43] and combined with the results of our analyses, permits the conclusion that training courses to increase cultural competence do indeed make a contribution to the recognition of discrimination. In addition, international literature recommends that staff training should not simply aim to increase cultural knowledge, but that course participants should also rehearse adequate reactions to acts of discrimination [7] or be provided with education in job training on how to become an ally when witnessing discriminatory acts [26]. It must be emphasized that a working climate that gives no space for affected staff to mention discrimination experiences and to discuss them can contribute to intensifying the problem [25].

### Limitations

One limitation of our work is that the cross-sectional study design cannot determine causality between the outcomes examined and associated characteristics, unlike longitudinal studies. In addition, despite recruiting across professions in two organizations, the response rate of just under six percent resulted in a small number of respondents. Since sociodemographic information on the total population of contacted staff was not available, non-responder analysis is pending. One can assume that overlooked and deleted email invitations have contributed to the low response rate. We further point out that the survey was in German only, resulting in potential bias in the sense of being less representative of migrant staff with an insufficient command of German. Finally, we measured respondents’ subjective perceptions of discriminatory acts and ascribed reasons which is increasingly encouraged in public health research to capture the discrimination of persons independently from allegedly objective categories, such as migration background [4]. However, our approach failed to distinguish discrimination experiences further regarding their frequency and exact circumstances of the occurrence.

## Conclusions

Hospital staff in the German healthcare system often experience discrimination related to a range of characteristics. Previous institutional responses to these occurrences were mostly insufficient and need to be developed. However, individualizing the response to discrimination experiences seems to represent an unsatisfactory approach. Various forms of cultural training courses can comprise one step toward reducing inter-staff discrimination, but do not represent an adequate stand-alone response to discrimination in healthcare. Even though individual institutions cannot annul social dynamics and relations, they should consider and develop anti-racist instruments on all institutional levels, particularly in view of increasing diversity in clinical teams. The findings of our study can give an impulse for further studies on discrimination and discriminatory structures in the German-speaking healthcare system with a specific focus on migration and ethnicity. Future studies should further pursue indications of intersectional differentiation of discrimination experiences in healthcare professions.

## Supplementary Information

**Additional file 1.** Selection of items used in the online survey.

## Data Availability

The data set generated and analysed during the current study is not publicly available due to a missing statement in participants’ information regarding data sharing but an anonymized data set is available from the corresponding author on reasonable request.

## Abbreviations

COVID-19: Coronavirus disease 2019
IMAZ e.V.: Interkulturelles Migrantenzentrum e.V.
QR: Quick response
