# Supplementary material for: Observed and personally experienced discrimination: findings of a cross-sectional survey of physicians and nursing staff
Source: Hum Resour Health. 2022 Dec 9;20:83. doi: 10.1186/s12960-022-00779-0 (PMC9733037; doi:10.1186/s12960-022-00779-0)
Supplement: Supplementary file 1 — Additional file 1. Selection of items used in the online survey. [file 12960_2022_779_MOESM1_ESM.docx]

# Selection of items used in the ToP online survey

Question 1: Where do you work?

1. Organization 1
2. Organization 2
3. Other 🡪 *End of questionnaire: Thank you for participating! Our survey is aimed exclusively at members of Organization 1 or Organization 2.*

Question 2: Which professional group do you belong to?

1. Physician
2. Nursing staff, nursing management and similar
3. Other professional group 🡪 *End of questionnaire: Thank you for participating! Our survey is aimed exclusively at physicians and nursing staff.*

Question 3: Is your employment contract temporary?

1. Yes
2. No

Question 4: Do you work full time?

1. Yes
2. No

Question 5: In your estimation, how big is the proportion of staff with a migrant background in your department? *(Immigrant or at least one immigrant parent)*

- *Open ended: proportion in %*

Question 6: In your estimation, how big is the proportion of patients with a migrant background in your department? *(Immigrant or at least one immigrant parent)*

- *Open ended: proportion in %*

Question 7: I have witnessed discrimination in our department

1. Yes 🡪 *Filter: Question 8, Question 9, Question 10*
2. No

Filter Question 8: Who was discriminated against? *Multiple answers possible*

1. Patient
2. Colleague
3. Superior
4. Other, namely *(open ended) …………………………………………………………………*

Filter Question 9: Why was this person discriminated against? *Multiple answers possible*

1. External appearance (skin color, appearance)
2. Religion/ ideology
3. Ethnic background/ nationality
4. Language
5. Age
6. Gender
7. Other *(open ended) ……………………………………………………………………………..*

Filter Question 10: Who carried out the discrimination? *Multiple answers possible*

1. Patient
2. Colleague
3. Superior
4. Other, namely *(open ended) …………………………………………………………………*

Question 11: I have personally experienced discrimination in our department

1. Yes *🡪 Filter: Question 12, Question 13*
2. No

Filter Question 12: Who discriminated against you? *Multiple answers possible*

1. Patient
2. Colleague
3. Superior
4. Other, namely *(open ended) …………………………………………………………………*

Filter Question 13: Why were you discriminated against? *Multiple answers possible*

1. External appearance (skin color, appearance)
2. Religion/ Ideology
3. Ethnic background / nationality
4. Language
5. Age
6. Gender
7. Other *(open ended) ……………………………………………………………………………..*

Question 14: Please state your gender

1. Male
2. Female

Question 15: Please state your date of birth (*year, e.g. 1980*)

- Year ………………………………

Question 16: Please state your country of birth *Please name the country to which your place of birth belonged at the time of your birth*.

1. Germany
2. Other country 🡪 namely *(open ended)…………………………..….*

Question 17: Please state your father’s country of birth *Please name the country to which his place of birth belonged at the time of his birth.*

1. Germany
2. Other country 🡪 namely *(open ended)…………………………..….*

Question 18: Please state your mother’s country of birth *Please name the country to which her place of birth belonged at the time of her birth.*

1. Germany
2. Other country 🡪 namely *(open ended)…………………………..….*
